# Nutrition of Pregnant and Lactating Women in the First 1000 Days of Infant

**DOI:** 10.3390/healthcare10010065

**Published:** 2021-12-30

**Authors:** Claude Billeaud, Juan Brines, Wafae Belcadi, Bérénice Castel, Virginie Rigourd

**Affiliations:** 1 Neonatology & Nutrition, CIC Paediatrics INSERM 1401 CHU, 33076 Bordeaux, France; 2 Department of Pediatric, Obstetrics and Gynecology, Faculty of Medicine, Valencia University, 46010 Valencia, Spain; juan.brines@uv.es; 3 CIC Paediatrics INSERM 1401 CHU, 33076 Bordeaux, France; wafae.belcadi@chu-bordeaux.fr (W.B.); berenice.castel@orange.fr (B.C.); 4 Neonatology and Director of “Lactarium de l’ile de France” Necker-Enfants Malades Hospital, 75015 Paris, France; virginie.rigourd@nck.aphp.fr

**Keywords:** breastmilk, human milk, pregnancy nutrition, lactation nutrition, lipids

## Abstract

Nutrition for pregnant and breastfeeding women is fundamental to the development of the child in its first 1000 days and beyond. To evaluate the adequacy of this nutrition, we have relied on historical dietary surveys and on personal French studies (4 studies from 1997 to 2014) involving dietary surveys over 3 days (3D-Diet). Furthermore, our team specialized in lipids has measured the fatty acids of breast milk, which reflect the dietary intake of lipids, from breast milk (1997–2014) and from the lipids of cord blood and maternal fat tissue, in 1997. According to our results, pregnancy needs require an additional 300 Kcal, but surveys show a bad equilibrium of macronutrients: an excess of proteins of fetus [17% of total energy intake (TEI) vs. 15%], excess of fats (45% vs. 35%), excess of saturated fatty acids (SFA), not enough polyunsaturated fatty acids (PUFA), particularly omega 3, and a deficit in carbohydrates (45% vs. 55%). There is also a deficiency in calcium, iron, magnesium, zinc, and vitamins D, B6, B5, and folates. Breast milk adequately provides all the macronutrients necessary for the growth of the child. Proteins and carbohydrates vary little according to the mother’s diet; on the other hand, its composition in lipids, trace elements, and vitamins is highly variable with the mother’s diet of breast milk. In our study in 2014, in 80 participants, the diet was low in calories (1996 Kcal vs. 2200 Kcal RDA), normoprotidic, normolipidic, but low in carbohydrate, especially polysaccharides. We note a very insufficient intake of fish and dairy products, and therefore calcium, but also magnesium, zinc, iron, and vitamins D, E, B6, and folate. Consequently, if the mother does not achieve a diet adequate to her needs during pregnancy and breastfeeding, it will be necessary to resort to medicinal supplements in minerals, trace elements, vitamins, and omega 3.

## 1. Introduction

The supply of nutrients to the fetus and the infant during the first 1000 days of the child’s [1] life, from conception to the end of the second year, depends on the supply of nutrients from the diet of the pregnant and breastfeeding woman.

Therefore, the mother’s diet and lifestyle before and during pregnancy and lactation constitute a determining factor in the infant’s health that can be projected on that of the child and future adult [2].

**Figure 1:**
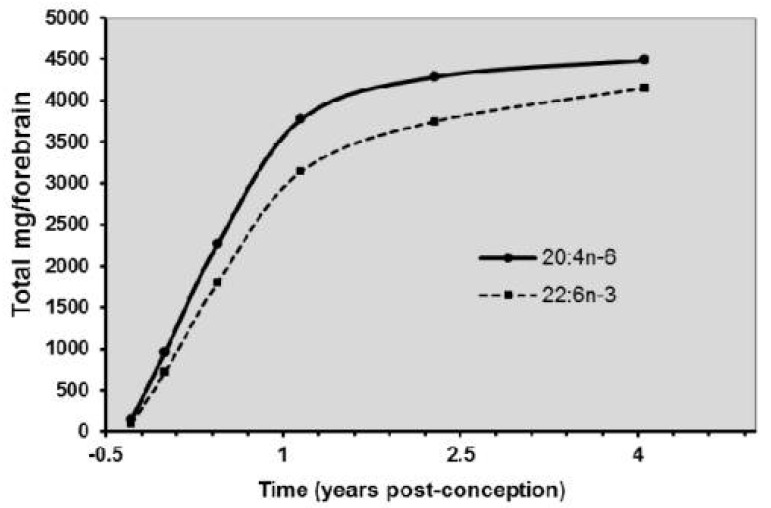
Accretion of docosahexaenoic acid (DHA-22:6n-3) and arachidonic acid. (AA-20:4n-6) in the brain of deceased children during the first years of life.

Without diminishing the importance of the macronutrients (proteins, lipids, and carbohydrates), in recent decades, research on human milk and infant nutrition has been mainly focused on the role of some essential fatty acids, vitamins, and trace elements. This interest has been greatly motivated by the demonstration of the causal relationship between folic acid deficiency in early pregnancy and neural tube defects in the fetus [3], or the function that long-chain essential fatty acids (omega 6 and omega 3 families) play for neuro-sensory development during pregnancy and lactation (Figure 1).These relationships between some nutrient deficiencies and pathologies of the fetus and infant have directly raised questions regarding the possibility of prevention through proper supplementation [4].

In this line, our research team has been particularly interested in the lipids in breast milk that reflect the nature of the lipids in the mother’s diet. The supplementation with the very long-chain fatty acid omega-3 docosahexaenoic acid (DHA) not only promotes brain and retinal development but also plays a role in improving preterm birth [4]. The aim of this paper is to evaluate the adequacy of the nutrition intake during pregnancy and lactation with recommended Diet Intake (RDI). We have relied on historical dietary surveys and on personal French studies (4 studies from 1997 to 2014) and one European Multicenter Study (2016–2020).

## 2. Material and Methods

We have compared food consumption by 3D-Diets on the one hand, and the RDI from the nutrition committees on the other to try to answer the question of supplements during pregnancy [5,6].

We have relied on historical dietary surveys, on personal French studies (4 studies from 1997 to 2014) and one European Multicenter Study (2016–2020). We have used a few demonstrative dietary surveys: an American survey: W.I.C. (Women, Infants and Children; *n* = 500,000 women) and two French surveys: one reported by Papoz [7] (*n* = 534 women); the other by Lecerf (*n* = 50 women). We compare the results of these different surveys with the RDI of the French Nutrition Committee (CNERMA).

In addition, we have used the experience of our research group conducting 4 clinical studies on 3-day dietary surveys from 1997 to 2014 (1997: 18 samples of milk; 2007: 142 samples of milk; 2012: 22 samples of milk and 2014: 80 samples of milk). We studied diets of lactating women between 2- and 4-months post-partum (Mature breast, milk samples collected in the morning). The FAs composition of breast milk was determined by direct transesterification and analyzed by FID-GPC. The data were compared by ANOVA and/or Kruskall and Wallis test.

In addition, our laboratory team has studied lipids measuring fatty acids in the blood of the mother and the newborn at birth cord, as well as in the adipose tissue on the cesarean scar.

Given that energy intake varies a lot according to BMI, a first visit at the beginning of pregnancy was considered to evaluate the woman’s nutrition status and performed.

## 3. Results and Comments

We will treat separately the data referring to the period of pregnancy and lactation.

### 3.1. Pregnant Women Nutrition 

#### 3.1.1. Energy Needs

The energy needs of pregnant women are estimated to 80,000 Kcal per 250 days, so 2300 Kcal per day [8].

Our estimate regarding additional nutrient needs per day according to the gestation period are as follows: during the first trimester, no caloric supplement; during the second trimester, 200 Kcal; and during the third trimester, 450 Kcal. Regarding the supplement needs according to the gestation period, Koletzko considers that they should not exceed 10% of the total energy [9].

#### 3.1.2. Macronutrients

The comparison between pregnant and non-pregnant macronutrient needs are the same and there is an excess of total fats and proteins, but not enough carbohydrates (Table 1) and energy. Indeed, the protein intake in surveys represents 15% of the total energy intake (TEI) versus 13% of the TEI recommended, 85 g/d versus 60 g/d (RDI). Fats consumed 42–43% of the TEI versus 35% (RDI) and carbohydrates are insufficient, 42% of TEI versus 55% (RDI.) (Table 1).

The comparison between the diet’s surveys and RDI shows a deficit in energy and carbohydrates.

#### 3.1.3. Proteins

Protein should represent 13% of the total energy intake, 1.1 g/kg/d [4]. In fact, on average, mothers consume 18% of TEI in our survey in 1997 and 17% in 2014. Moreover, we should provide 2/3 of animal proteins of good biological quality and 1/3 of vegetable proteins. The problem arises with vegetarians who need to sort out vegetables’ rich in essential amino acids and compensate for deficits in micronutrients and vitamins [4].

#### 3.1.4. Fats

We distinguish three types of fatty acids (FA): 1—saturated fatty acids (SFA) (no double bonds), 2—monounsaturated fatty acids (MUFA, 1 double bond), the most important of which is oleic acid, and 3—polyunsaturated fatty acids (more than two double bonds) which are the essential fatty acids, precursors of the omega 3 and 6 families: linoleic acid (LA) and omega 3 family: linolenic acid (ALA) which are converted in long chain polyunsaturated fatty acids (LC-PUFA: Arachidonic acid “AA” omega 6, and docosahexaenoic acid “DHA”, omega 3).

The intake of all types of fats varies with the mother’s fat diet: oils or meats and fish intake AA and DHA are presented in Table 2 and Table 3.

Meat, eggs, and fish bring Arachidonic acid. Oily fishes (Table 3), such as mackerel, 170 g twice a week brings DHA (350 mg/day). Fish, and mainly oily fish, intake much DHA. The Mackerel Fish is the richest in DHA [10]. Oily fish may contain traces of mercury but the impact on child health might be outweighed by the importance of DHA intake on neuro-sensory development. Among the oily fish low in mercury, sardines, herring, and salmon should be best [4].

There are many Long Chain Polyunsaturated fatty acids (LCPUFA) needs for the brain (Figure 1), as seen for the fetus from the 22nd week of the gestational age to 2- or 3-years postnatal age. The total need in preterm is about 45 mg/kg/d, at least, and up to 60 mg of DHA at birth [11]. The precursors (ALA and LA) of long-chain polyunsaturated fatty acids (LCPUFA) are poorly metabolized to LCPUFA (DHA and AA). The placenta compensates for this by a biomagnification phenomenon transferring more LCPUFA (DHA and AA) to the cord blood and therefore to the fetus than the precursors (ALA and LA) [11].

In the Adipose tissue of pregnant women in a 1997 study [12]: there is a major content of Saturated FA (32%) and Monounsaturated FA (51%). There is a bad ratio of Linoleic/linolenic acid 32, an excess of omega 6 (16%), and a lack of omega 3. Therefore, we advise to intake more precursor of omega 3 (ALA). Polyunsaturated fatty acids are essential, although there is a sufficient intake in fat too rich in linoleic acid provided by olive oil and deficient in linolenic acid. The optimal Linoleic/linolenic ratio is 6–7 so that the precursors are more easily transformed into long-chain fatty acids [13].

DHA and AA are essential for brain and retinal development and a supplementation in DHA (200–350 mg) is necessary during pregnancy and lactation [14].

Pregnant women find these long-chain fatty acids in their food:

She finds ALA, EPA, and DHA in oily fish (salmon, mackerel, sardines, tuna), lean fish and AA in eggs, meat, and offal (Table 2). Varying the oils (rapeseed mainly instead olive) and eating oily fish, eggs, and meat are sufficient for a good lipid nutritional balance [10].

*Trans fatty acids (TFAs)* are harmful polyunsaturated fatty acids. Recent studies have suggested that *TFAs* compete cis-PUFA metabolism: our study [12] is according to the study performed by Koletzko [15], who reported that in premature infants, the level of plasma *elaidic acid* is inversely correlated to long-chain PUFA levels (LA). An inverse correlation is also observed between *TFA* levels and birth weight. This suggests that an exposure to high levels of *TFAs* during pregnancy may impair fetus growth. The *TFAs* are found in bad fats (shortenings) and white bread and pastries made with bad fats. The most elevated is 17% of Total Fatty Acids in Canada and the USA. In France, 2% in 1997, and only 1% in 2014, after improving the margarine quality in France [16].

#### 3.1.5. Mineral Salts and Trace Elements

It is possible to balance the need for minerals, trace elements, and vitamins with a diet as previously defined. As the recommended calcium requirement is 1000–1200 mg/d, a small supplement of 200 mg of calcium is needed [17] (Table 4).

This can easily be met if we drink more milk; however, this will provide more protein and more saturated fat. There is a slight deficit (compared to the RDI) in magnesium (150 mg/d) and iron (15 mg/d) if we consider that iron requirements are 30 mg/d or more if there is anemia in pregnant women [18]. For zinc, there is a shortfall of 10 mg, the usual recommended requirement is 19 mg, and the diet provides only 4–5 mg. In addition, the Iodine deficiency must be corrected [19]. The WHO recommends a daily intake of 250 μg iodine for pregnant women.

#### 3.1.6. Vitamins

A usual diet provides only 3 micrograms of vitamin D, and the requirement is 10 mg–12 mg to ensure the security needs during pregnancy, depending on the amount of sunshine. Northern countries need a daily intake of 5 mg/day, which is surprising [20], and for Southern countries the supplementation is not systematic, or a minimum of 5 mg.

*Group B vitamins*: while vitamins B_1_ and B_6_ are moderately deficient, if we consider that folate (vitamin B_9_) requirements are 500 µg, there is a risk since the average intake is 255 µg. Iron, cobalamin, and folate deficiencies must be associated in pregnancy anemia [3].

In total, with a balanced diet, the needs in proteins, lipids, carbohydrates, and energy are covered. On the other hand, the intake of calcium, iron, vitamin D, iodine, and folate remains insufficient (Table 5).

The micronutrients and vitamins intake are not always perfectly guaranteed. It seems necessary to take care of this and add a supplement. Most often, we use multivitamins and oligoelement supplements with no toxicity, except for vitamin A when we exceed 8000 ui/d, which could lead to heart and circulatory malformations, but retinol alone is teratogenic but not the carotenes [21].

If the mother does not eat sufficiently oily fish, she must take a supplement of 200 to 350 mg of DHA.

### 3.2. Lactating Mothers Nutrition

Breast milk adequately provides all the macronutrients necessary for the harmonious growth of the child. The composition of breast milk in proteins and carbohydrates varies little according to the mother’s diet. On the other hand, its composition in lipids, trace elements, and vitamins is very variable with the mother’s diet. These results come from French and European multicenter studies [10,22,23,24] and American RDI [25,26,27], and also from our French studies 1997–2014 and from the Multicentric European Study during 2016–2020: the ATLAS [28,29] and the Doctoral Thesis on Nutritional Sciences of one of the authors [30].

#### 3.2.1. Energy

The diet of breastfeeding women in 1997 was hyperproteic (105 g/d vs. 80 RDI), hyperlipidic (130 g/d vs. 90 RDI), and often with an excess of saturated fatty acids (15% of energy vs. 12% RDI), a deficit in polyunsaturated fatty acids (5% vs. 7% RDI), insufficient in carbohydrates (285 g/d vs. 340 RDI), and hypercaloric at 2700 kcal.

In 2014, the 3-D-diets showed that it was normoprotein and normolipid, but hypocaloric (1992 kcal/d) compared to 2300 kcal (RDI) and to ATLAS (2044 Kcal/d, 87 g/d of proteins, 78 g/d of fats, 285 g/d of carbohydrates).

The newborn consumes about 750 g of breast milk, which would mean that the mother would need to produce this amount of milk with a supplement of 450 Kcal/d. However, considering the stocks built up during pregnancy, the real supplement is between 70 and 200 Kcal/d [26].

#### 3.2.2. Proteins

The requirements are covered by the normal diet; there is even a tendency to overconsume. The recommended protein intake during pregnancy is 1.1 g/kg/d, and 0.8 g/kg/d [4] during lactation. These values have been revised downwards due to the risk of obesity in the child if too much protein is consumed during the first 1000 days of life. In terms of quality, the intake of animal proteins with a high biological value must represent at least 2/3 of the intake, which is the case in the French surveys; the remainder can be made up of vegetable proteins. Animal proteins are represented by dairy proteins on the one hand, but also by meat, fish, and eggs.

Vegans have proteins with a bad biological value and a deficit of vitamin B_12_, D, and microelements requiring a supplement [3].

This dietary behavior leads to an insufficient intake of calcium and an excess of animal fats. Milk and dairy products are particularly useful in breastfeeding women to provide calcium (1/2 L = 600 mg), such as firm cheese (700 mg/100 gr). On the other hand, such as all foods of animal origin, they contain saturated fats. It is therefore recommended to use semi-skimmed milk or special milk for pregnant women (enriched with vitamins and trace elements).

#### 3.2.3. Carbohydrates

Overall, the breastfeeding woman consumes relatively few carbohydrates (285 g/d in French studies and 235 g/d in ATLAS vs. 340 RDI), (40% TEI vs. 53% RDI), and in terms of quality, there is an excess of “simple” sugars (mono-disaccharides) (110 g/d vs. 10% RDI, i.e., 60 g/d), and not enough polysaccharides (110 g/d) and fiber (11 g/d in French studies and 20.2 g/d in ATLAS vs. 25 g/d RDI).

In terms of foods, sweets and pastries are consumed in excess by more than half of the mothers. Bread (90 g/day vs. 250 g/day) and starchy foods (160 g/day) are avoided and lead to a deficit of fibers which participate to intake an important functional human milk oligosaccharides (HMO) and a deficit of slow sugars. The drink must be abundant (2 L + water from food), 2.6 L/d in our study in 2014 avoiding sweetened fruit juices (155 mL/d in our study vs. 100 mL desired).

#### 3.2.4. Fat’s Composition

Fat’s composition of breast milk varies greatly with the mother’s diet. A mother who is exclusively vegetarian will have a milk richer in fatty acids of the ω6 series (LA), whereas Eskimo women, consuming exclusively fish, will produce a milk very rich in fatty acids of the ω3 series (ALA, EPA and DHA).

#### 3.2.5. Essential Fatty Acids

Our team has undertaken the regular monitoring of dietary surveys (Table 6). In France, the monitoring was held regularly from 1997 until the ATLAS study in 2020 to see the effect of a national program advising a higher intake of omega 3. Since 1997, the French nutrition committees have recommended consuming more omega 3. We can see that between 1997 and 2014, ALA has increased significantly from 0.52% to 0.96%, DHA has increased but very slightly from 0.24% to 0.29% [24]. In the ATLAS study, we measured fatty acids in mature milks from seven European countries, including France (*n* = 85), the results of a publication accepted in the European Journal of nutrition (December 2021). The results are not different from the French results of 2014 [29].

In 2014 [10], we performed a nutritional intervention on a group of 80 breastfeeding women by giving mackerel 170 g twice a week, providing 350 mg/d of DHA and ALA, which respectively increased from 0.29% to 0.54%, and ALA from 0.96% to 2.15% while the LA/ALA ratio decreased from 10.73 to 5.5. We found that the AA line decreased from 0.36% to 0.33%. We recommend that breastfeeding women should consume 10% LA and 2–4% of total fatty acids (*TFA*) as ALA from 30 g/day of rapeseed oil and 25 g of margarine enriched with omega 3, and oily fish such as mackerel 170 g twice a week to increase DHA and 0.33–0.7% of AA from eating eggs and meat. In the ATLAS study, the FA intakes results were: SFA, 32 g/d or 41% of total fatty Acids (*TFA*); LA, 6.4 g/d i.e., 8.2% of *TFA*; ALA, 1 g/d i.e., 1.2% vs. 4% of *TFA*; EPA, 170 mg/d i.e., 0.2% of *TFA*; and DHA, 250 mg/d i.e., 0.3% of *TFA*. These results show an excess of SFA and an insufficiency PUFA (LA and ALA), but on the other hand, an intake of DHA at 250 mg, which is sufficient (with great variations according to the diet of each woman).

In 2018, another French Study [31] concluded, “Main results showed that mean daily intakes of n-3 PUFA were very low in this French woman population because no pregnant and lactating women met recommended dietary intakes (RDIs).”

#### 3.2.6. Minerals, Vegetables, Oligo-Elements, Vitamins

##### Vegetables

The category of vegetables and fruit is important for dietary balance as it provides minerals, vitamins, and fibers. We observed a low consumption of raw vegetables (20 g/d vs. 100 desired), vegetables (140 g/d vs. 250 desired), and fruit (160 g/d vs. 300 desired).

##### Minerals–Trace-Elements

The dietary behavior leads to an insufficient intake of calcium. Milk and dairy products are particularly useful in breastfeeding women to provide calcium (1/2 L = 600 mg), firm cheese (700 mg/100 g). On the other hand, such as all foods of animal origin, they contain saturated fats. It is therefore recommended to use semi-skimmed milk or special milk for pregnant women (enriched with vitamins and trace elements). Calcium intake is very important. In fact, the production of 800 mL of breast milk leads to an additional need of 200 mg, i.e., 1000–1200 mg/d. In 1997, our survey showed 6 times out of 18 there was an insufficiency of milk consumption and therefore of calcium, but also of Mg (250 mg/d vs. 480 mg/d RDI) and of Zinc (7 mg/d vs. 19 RDI). The Atlas study shows: (Calcium 957 mg/d vs. 1000–1200 mg RDI), Magnesium (322.2 mg/d vs. 480 mg RDI), Zinc (10.2 mg vs. 19 mg RDI), and Iron (16.4 mg vs. 9.5 mg RDI).

##### Vitamins

There is a real interest in suggesting vitamin supplements in view of the insufficiency of vitamin D (2 g/d vs. 10 RDI), vitamin E (7 mg/d vs. 12 RDI), B6 (1.5 mg/d vs. 2.5 RDI), and folates (138 g vs. 500 RDI). A nutritional supplementation improves the coverage of oligo-elements and vitamins. The Atlas study shows a deficiency in pantothenic acid, folate, vitamin C, vitamin A, and vitamin D (Vit D, 5.3 mg/d vs. 10 RDI) (Vit A, 819 mg/d) (Vit B_1_, 1.6 mg/d; vit B_6_, 2 mg/d; folates, 337.3 mg/d: vit C, 120.5 mg/d) (Table 7).

##### Proposal for a Typical Menu for Breastfeeding Woman

It is a balanced menu providing proteins in the form of dairy products (300 mL of semi-skimmed milk, 2 yoghurts, 1 portion of cheese), no more than 150 g of “lean” meat or 2 eggs or oily fish (twice a week), which represents 15% of the energy intake in the form of protein per day, and slow sugars will be provided by potatoes, bread, and cereals, to the detriment of rapidly adsorbed sugars.

What kind of oils? This is the originality of the diet that we recommended: little butter (10 g per day) and fats (30 g of rapeseed oil, i.e., 2 spoons, 25 g of margarine enriched in ALA, mackerel 170 g twice a week for DHA, eggs, and meat twice a week for AA), starchy, and bread (250 g each) per day. With these recommendations, the proper intake of vitamins and oligo-elements is not assured, so it is advisable to guarantee it through supplements.

## 4. Conclusions

It is true that the pregnant woman has spontaneously balanced her nutritional intake for the child she is carrying, since man has been able to perpetuate himself up to that point, but the micronutrients and vitamins intake were not always perfectly guaranteed; it is necessary to ensure this for the well-being of the fetus and the mother.

A new concept has appeared, still “in gestation”, that of “milk” or “nutritional supplement” for pregnant women. Milk is in fact an extremely practical food “vector” for adding micronutrient supplements in a harmonious way. It is also necessary to insist on the “variety” and “quality” of the diet, which guarantee a good nutritional balance [32].

In addition, it would seem desirable that every pregnant woman, especially if she belongs to a risk group (multiple or repeated pregnancies, vegetarians, teenagers, socio-economic problems, etc.), should be able to benefit from a consultation with a dietician from the “maternity ward” at the beginning of her pregnancy in order to determine for each of them a dietary pattern, and, if necessary, to prescribe appropriate nutritional supplements.

Obviously, the intake of alcohol and drugs, as well of medications contraindicated during pregnancy and lactation should be totally prohibited.

## Figures and Tables

**Table 1:** Survey and macronutrients/RDI.

Nutrients	W.I.C USA	LECLERC France	PAPOZ France	RDI	Needs
Energy Kcal/day	1512–2400	2233	2136	2300	+100–450
Protein g/day	68–110	85 g 15%	78 g 15%	60 g 13%	Excess Quality
Fats g/day		104 g 42%	103 g 43%	90 g 35%	Excess Quality
Carbohydrates g/day		235 g 42%	211 g 40%	340 g 55%	+100 Quality

**Table 2:** Fatty acid oils composition.

Oils	Peanuts	Rapeseed	Hazelnut	Olive	Grapeseed	Soja	Sunflower	Sunflower Oleic
Saturated FA SFA	48–66	55–62	24–32	61–80	14–22	17–26	15–25	75–83
Monounsaturated MUFA	49–68	56–65	25–33	32–81	15–23	18–27	16–26	75–84
Linoleic Ac C18:2 n-6	14–28	18–22	55–62	3–14	65–73	50–62	62–70	10–21
Linolenic Ac C18:3 n-3	<0.3	8–10	<2	<1	<0.5	4–10	≤0.2	≤0.3
Polyunsaturated PUFA	14–28	26–32	57–64	4–15	65–73	54–72	62–70	10–22

The composition of omega 6 and 3 oils is different: rapeseed, soybean, walnut, and hazelnut oils contain both ALA (ω3) and LA (ω6), whereas olive oil contains no omega 3 (no ALA).

**Table 3:** Fish fatty acid composition.

Oily Fish	Arachidonic Acid (AA) (mg/100 g)	Eicosapentaenoic Acid EPA (mg/100 g)	DHA (mg/100 g)
Mackerel	70	1020	1940
Sardine	-	1250	1790
Salmon	41	527	842
Albacore Tuna	42	562	313
**Lean Fish**	**Arachidonic Acid (AA) (mg/100 g)**	**EPA (mg/100 g)**	**DHA (mg/100 g)**
Wild Sea Bar	-	220	293
Cod	<10	77	194
Dab	<14	102	189
Sole	<11	19	81

The table shows different contents in omega 3 (ALA, EPA, DHA) between oily and lean fishes.

**Table 4:** Surveys minerals and/RDI.

Nutrients	W.I.C USA	LECLERC France	PAPOZ France	RDI	Needs
Calcium mg/d	668–1670	975	869	1000	200
Magnesium mg/d	187–269	339	260	480	300
Iron mg/d	11.4–17	13.7	12.4	30	30
Zinc mg/d	6–12			19	15

The table shows a deficiency in pregnancy in Calcium, Magnesium, Iron, and Zinc.

**Table 5:** Survey vitamins and RDI.

Nutrients	W.I.C USA	LECLERC France	PAPOZ France	RDI	Needs
Vit D mg	3–5	3.4		10	+10
Vit B_1_ mg/day	1.2–1.7	1.3	1.3	1.5	+2.5
Vit B_6_ mg/day	0.6–2.1	1.6	1.7	2.5	+2
Folates (B_9_) mg/day	144–243	255	53	500	+300

The table shows a deficiency in pregnancy in vitamins D, B_1_, B_6,_ and folates.

**Table 6:** Fatty acids evolution in mature human milk from 1997 to 2020 in FRANCE [10,24,29].

Fatty Acids %	1997 PhD *n* = 18	2007 JFRN *n =* 142	2012 Barcelona *n* = 22	2014 [10] (1) PHRC ω3 *n* = 80	2014 [10] (2) PHRC ω3 *n* = 80	2020 [29] ATLAS *n* = 85
ALA	0.52 (0.2) ^b^	0.83 (0.14) ^b^	0.86 (001)	0.96 (0.50) ^a^	2.15 (0.74) ^a^	0.93 (0.26)
LA	13.33 (19.62) ^b^	11.14 (10.24)	9.27 (0.34) ^b^	10.03 (3.0)	10.77 (2.11)	10.51 (1.46)
LA/ALA	27.63 ^b^	13.42 ^b^	10.77	10.73 ^a^	5.54 (2.11) ^a^	11.30
EPA	0.08 (0.003)	0.07 (0.002)	0.06 (0.0001)	0.09 (0.05) ^a^	0.17 (0.01) ^a^	0.09 (0.03)
DHA	0.26 (0.01)	0.24 (0.01)	0.24 (0.003)	0.29 (0.16) ^a^	0.56 (0.40) ^a^	0.33 (0.11)
AA	0.38 (0.05)	0.40 (0.01)	0.39 (0.001)	0.36 (0.07) ^a^	0.33 (0.22) ^a^	0.43 (0.07) ^a^
AA/DHA	1.46	1.67	1.63	1.24 ^a^	0.82 (0.15) ^a^	1.52
SFA	48.05 (27.67)	47.50 (27.98)	48.84 (4.28)	46.71 (4.38)	43.76 (3.88)	43.96 (0.59)
MUFA	32.80 (13.39)	37.76 (15.84)	38.60 (2.62)	39.72 (3.12)	40.60 (7,63)	43.1 (3.79)
PUFA	15.35 (21.72)	13.45 (11.83)	11.64 (0.45)	12.54 (3.3) ^a^	14.70 (2.7) ^a^	12.95 (2.6)
*TFAs*	2.10 (0.62) ^b^	1.30 (0.36) ^b^	0.92 (0.09) ^b^	1.03 (0.29)	0.94 (0.27)	-

**^a, b^** is statistical difference *p* < 0.05. ^a^: is significant difference in interventional nutrition between column PHRC w3 (1) and (2). ^b^: is significant differences in spontaneous nutritional evolution with time.

**Table 7:** Pregnancy and lactation recommended nutriments. ANSES. 2019 [23].

Nutrients	Pregnancy		Lactancy	
	Intake	RDI	Intake	RDI
Proteins % TEI	18	13	17	20
Fats % TEI	44	35	43	40
SFA % TEI	13	≤	15	12
MUFA% TEI	13	15	8	20
ω6 PUFA % TEI	4.1	=	3.9	4
ω3 PUFA % TEI	0.4	=	0.3	1
ω6/ω3	10	≤	13	5
Carbohydrates % TEI	38	40	40	55
Simple carbohydrates % TEI	14	≤	18	10
Fibers (g)	12	25	20	30
Alcohol (g)	3	=	5	0
Calcium (mg)	900	1000	800	1000
Magnesium (mg)	200	400	250	390
Iron (mg)	14	30	9.5	10
Zinc (mg)	5	14	9	19
Vitamin D (µg)	2.4		1.9	10
Vitamin B6 (mg)	1.5	=	1	2
Vitamin B9 (µg)	154	=	242	400

## Data Availability

The data presented in this study are available on request from the corresponding author. The data are not publicly available due to privacy and ethical restrictions.
